# Optical Coherence Tomography Angiography Biomarkers of Retinal Thickness and Microvascular Alterations in Sjogren's Syndrome

**DOI:** 10.3389/fneur.2022.853930

**Published:** 2022-03-08

**Authors:** Ren Liu, Yan Wang, Qiuyu Li, Qiang Xia, Tian Xu, Ting Han, Shuang Cai, Shuilin Luo, Rui Wu, Yi Shao

**Affiliations:** ^1^Department of Rheumatology, The First Affiliated Hospital of Nanchang University, Nanchang, China; ^2^Department of Ophthalmology, The First Affiliated Hospital of Nanchang University, Jiangxi Province Ocular Disease Clinical Research Center, Nanchang, China

**Keywords:** retinal thickness, Sjogren's syndrome, optical coherence tomography angiography, biomarker, vascular density

## Abstract

**Purpose:**

To investigate the differences of retinal thickness (RT) and superficial vascular density (SVD) between patients with Sjogren's syndrome (SS) and healthy controls (HCs) using optical coherence tomography angiography (OCTA).

**Methods:**

Individuals with SS and healthy controls were enrolled (*n* = 12 per group). An en-face OCTA scan was performed on each eye. Images were segmented into 9 subregions and macular RT and SVD were measured and compared between the 2 groups.

**Results:**

Visual acuity (VA) differed significantly between patients with SS (24 eyes) and controls (24 eyes) (*p* < 0.001). In patients with SS, inner RT was reduced in the inner superior region, outer RT was reduced in the outer nasal (ON) region, and full RT was reduced in the ON region compared with the control group (*p* < 0.05). RT was negatively correlated with serum IgG level in the outer and full retina at ON regions (*p* < 0.05). SVD in the inner nasal, ON, and inner temporal regions was significantly lower in patients with SS than in control subjects (*p* < 0.05). SVD was positively correlated with full RT in the ON region in patients with SS (*p* < 0.05). The areas under the receiver operating characteristic (ROC) curves for the diagnostic sensitivity of outer RT and full RT in the ON region for SS were 0.828 (95% *CI*: 0.709–0.947) and 0.839 (95% *CI*: 0.715–0.963), respectively.

**Conclusions:**

In patients with SS, retinal thinning in the macular area—which affects vision—can also reflect the severity of dry eyes in SS and has clinical value for assisted imaging diagnosis.

## Introduction

Sjogren's syndrome (SS) is a long-term diffuse connective tissue disease. The global prevalence rate is 61 people per 100,000 (1). The male-to-female ratio is between 1:9 and 1:10, and most patients are 30–60 years of age (2). The clinical manifestations of SS range from exocrine to systemic with multiple extraglandular involvement (3). Although SS is associated with a lower incidence of visceral damage than systemic lupus erythematosus (SLE), the degree of disability is similar (4). Patients with SS often have physical and psychological disorders that reduce the social functioning and quality of life (5, 6). The symptomatology of SS overlaps with that of other systemic diseases, drug reactions, and viral infections. Thus, SS diagnosis and treatment represent a clinical challenge. In fact, the two-thirds of patients with SS are not correctly diagnosed initially (3, 7) and a diagnosis can take up to 10 years from initial symptom onset (8).

Since its discovery in the 1930s, the classification of SS has been continually updated. SS is suspected when the following symptoms are present: (1) constant and annoying dry eyes persisting for at least 3 months; (2) continual foreign body sensation in the eyes; (3) requiring the use of artificial tears ≥3 times a day; (4) feeling thirst every day for more than 3 months; and (5) a need to drink water to swallow solid food (9). The European League Against Rheumatism (EULAR) and American College of Rheumatology (ACR) issued classification standards for SS that included clinical and immune evaluations, with less emphasis placed on the eyes (10).

Among patients with SS, the incidences of xerophthalmia and thirst—the most common disease symptoms—were 44% and 39%, respectively (11). However, a study with a large sample size found that dry eye was much more common than dry mouth (12). Patients with SS often have ocular surface inflammation, such as eyelid swelling and corneal ulcer (8, 13–15); and ocular lesions in other parts of the eye such as optic neuritis (8), uveitis (16), and scleritis (17) have also been reported. Over one-third of patients with SS have extraglandular ocular manifestations and when these interfere with vision, patients are more likely to have fatal complications than those without such lesions (8). In patients with systemic autoimmune disorders, the choroid and retina may not only reflect ocular diseases but also indirect ocular injury (18). In SLE, retinopathy is an accurate reflection of disease activity in both recessive and dominant cases, and SLE patients with related retinopathy have a significantly lower survival rate than those without retinopathy (19). In SS, the significance of retinal changes is worth exploring.

There is currently no single clinical index or gold standard assay for the diagnosis and/or differentiation of SS, although a lip biopsy is useful (20). However, the invasiveness of the procedure precludes regular follow-up and monitoring. The main measure of disease activity in SS is the EULAR Patient Report Index (21), which includes just one eye-related assessment item. Optical coherence tomography angiography (OCTA) is a novel *in vivo* imaging technique that provides detailed morphologic and quantitative information on microvascular changes in the eye (22). OCTA can be used to show the microvascular structure in the macula and optic disk (23, 24), which can facilitate the diagnosis of diseases, such as Alzheimer's disease (25), diabetes (26), multiple sclerosis (27), and thyroid-related ophthalmopathy (28).

To date there have been no studies that have applied OCTA to the diagnosis of SS. In this study, we investigated the ocular status of patients with SS, and used OCTA to assess retinal thickness (RT) and vascular density (VD) in patients with SS compared with healthy controls.

## Materials and Methods

### Subjects

This cross-sectional study was conducted at the Department of Ophthalmology and Rheumatology of The First Affiliated Hospital of Nanchang University (Nanchang, China) in 2020. Patients diagnosed with SS were recruited from the Outpatient Department of Rheumatism Immunology; and sex- and age-matched healthy normal subjects were recruited from the Ocular Disease Clinical Research Center. An ophthalmologist from the Medical Center evaluated the absence of abnormalities in the eyes of these subjects through clinical examination and OCTA imaging. All subjects were examined by the same retina specialist.

### Inclusion and Exclusion Criteria

All patients were women and met the 2002 American European Consensus Group classification criteria for SS (9). The patients ranged in age from 18 to 66 years. None of the patients showed symptoms or the signs of retinal vasculitis, choroiditis, or optic neuritis. Patients with chorioretinopathy induced by hydroxychloroquine (HCQ) were excluded.

Other exclusion criteria were as follows: (1) autoimmune diseases other than SS; (2) systemic diseases, such as endocrine, cardiovascular, or nervous system disease affecting the eye or optic nerve; (3) retinopathy or choroidal disease (e.g., arteriovenous disease, glaucoma, or high intraocular pressure (IOP); (4) any history of eye surgery or trauma or eye tumor; (5) contraindications, allergies, or intolerance to local anesthetics or mydriatic drugs; (6) diseases that could affect fundus imaging; and (7) pregnant or lactating women.

### Ethical Considerations

The study was conducted in accordance with the Helsinki Declaration and was approved by the hospital's ethics council (cdyfy2018026). Before signing the informed consent statement, all participants fully understood the research methods and objectives.

### Clinical Examinations

All subjects underwent the following clinical and ophthalmic examinations: (1) Immunological information, such as antinuclear antibody, anti-SS-A antibody, anti-SS-B antibody, IgG, and complement and salivary gland biopsy findings (29); (2) analysis of C-reactive protein (CRP) level and erythrocyte sedimentation rate to assess the patient's inflammatory status; (3) EULAR SS disease activity index for disease activity (ESSDAI) (21); (4) examination of mental state using the Hospital Anxiety and Depression Scale (HADS) (30); (5) ocular measurements, such as visual acuity (VA) (Snellen chart), IOP (Goldmann tonometry), spherical equivalent, and keratoconjunctivitis sicca; and (6) OCTA.

### Ocular Measurements

Tear breakup time (BUT): apply fluorescein sodium evenly on the ocular surface, observe the first tear film rupture point under cobalt blue light, and calculate the tear film rupture time. Less than 10 s is positive (31).

Ocular staining score (OSS): corneal fluorescein staining and conjunctival lysamine green staining were used for comprehensive evaluation. The ocular surface of each eye was divided into three parts: nasal conjunctiva, cornea, and temporal conjunctiva. The nasal and temporal conjunctiva were scored according to the number of conjunctival spots in the palpebral fissure area. An OSS score ≥3 was positive (9).

Schirmer's test (SIT): fold one end of 5 mm × 35 mm filter paper into right angle, put it into conjunctival sac after disinfection. The soaking length of filter paper is normally 15 mm/5 min and <5 mm/5min is positive (9).

Tear meniscus height (TMH): infrared light was used to focus and guide patients to blink. After white light shooting, the software (Keratograph 5M) was used to measure and record (32).

### Optical Coherence Tomography Angiography

We used an RTVue Avanti XR system (Optovue, Fremont, CA) to perform OCTA imaging to simultaneously display retinal cross section and microvessels. The scanning speed is set as 70,000 a-scans/s, the axial resolution is 5 mm, the horizontal resolution is 22 μm, the central wavelength is 840 nm, and the bandwidth is 45 nm. The imaging time was 3.9 s. Five angiography was performed in 3 mm × 3 mm scanning mode, focusing on the fovea along the *X*-axis (216 A scans in total) and along the *Y*-axis (216 grating positions for each scan). At 216 y positions ×5 positions, we captured 1,080 B scans at 270 frames/s (33). By 4 volume scans, OCTA images of 3 mm × 3 mm were obtained: a total of 933,120 A scans (2 horizontal scans and 2 vertical scans). A 3 mm × 3 mm en-face OCTA angiographic image was calculated for each eye.

**Figure 1:**
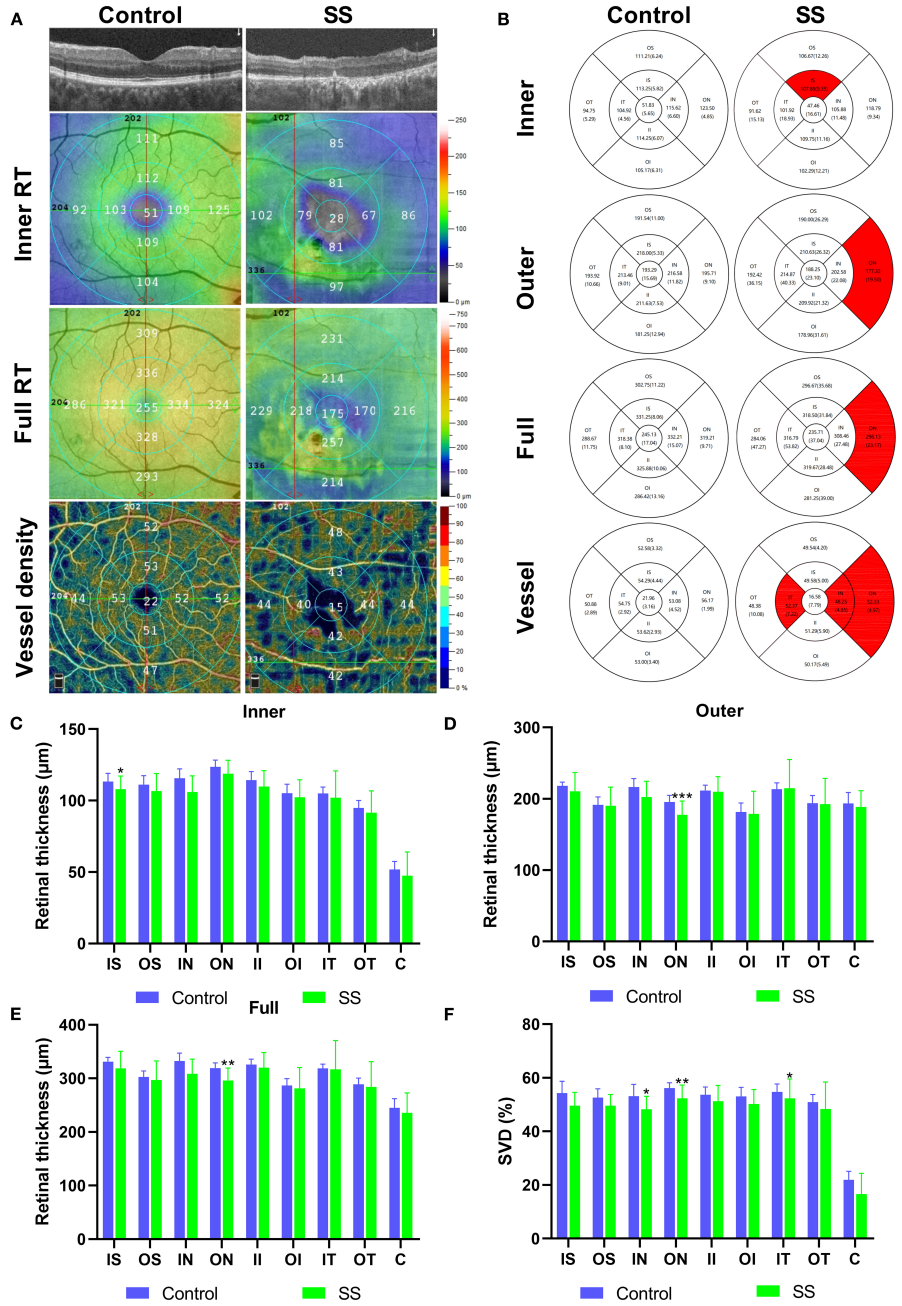
The OCTA images and RT and SVD analysis of control and SS groups. **(A)** Cross-sectional view of RT in SS and health group used OCTA. The inner RT, full RT and SVD were measured by ETDRS. **(B)** The results of inner RT, outer RT, full RT and SVD in the SS and healthy group were compared. **(C–E)** Analysis of RT results in the SS and healthy group. The vertical coordinate is the value of RT, and the horizontal coordinate is the retinal subregions. **(F)** Analysis of SVD results in the SS and healthy group. The vertical coordinate is the value of SVD, and the horizontal coordinate is the retinal subregions. OCTA, optical coherence tomography angiography; SS, Sjogren's syndrome; RT, retinal thickness; SVD, superficial vessel density; ETDRS, early treatment of diabetic retinopathy study; IS, inner superior; OS, outer superior; IN, inner nasal; ON, outer nasal; II, inner inferior; OI, outer inferior; IT, inner temporal; OT, outer temporal; C center. * *p* < 0.05; ** *p* < 0.01; and *** *P* < 0.001.

After scanning, each retina was segmented into nine early treatment diabetic retinopathy study (ETDRS) subregions, composed of three concentric round (0.5, 1.5, and 3 mm in radius), and their thickness was analyzed. Each story of the retina covers: (1) inner retina: from internal limiting membrane (ILM) to inner plexiform layer (IPL); (2) full retina: from ILM to retinal segment epithelium (RPE). We defined outer RT as the difference between full RT and inner RT. The vascular perfusion area as a percentage of the measured area was vascular density. Using the threshold method, vascular density was reckoned by creating two-dimensional en-face images of superficial retina (the layer between the vitreous retinal interface and the front boundary of the ganglion cell layer). Determine the value of the image block and assign it to each pixel perfusion (1) or background (0). The average value of the skeleton plate in the region of interest was scaled based on the pixel size (512 pixels/3 mm) to calculate vascular density from the center of the macula to the edge of the 3 mm × 3 mm brightness gradient image. Macular RT and superficial (S) VD were measured. All subjects used the right eye first. Data from the left eye were flipped to obtain a mirror image of the right eye. Left and right eye data were averaged and analyzed together (Figure 1A).

### Statistical Analysis

The data were analyzed using SPSS version 22.0 (IBM Corp, Armonk, NY, USA) and GraphPad Prism version 8 (La Jolla, California, USA) are reported as mean ± SD. Independent-samples *t*-test, chi-square test, and Fisher's exact test were used to compare data between groups. The generalized estimation equation was employed to compare RT and SVD between SS eyes and control eyes and data were adjusted for known confounding variables to explain interocular correlation within subjects. The relationship between RT and systemic and ocular variables was analyzed using univariate and multivariate regression analyses. A linear correlation analysis was conducted between RT (full thickness, inner layer, and outer layer) and SVD in each group. To analyze the distinction between healthy and SS subjects, receiver operating characteristic (ROC) curves for RT (full, inner and outer) and SVD were plotted. The value of *p* < 0.05 was deemed statistically significant.

## Results

### Subjects

There were 12 cases (24 eyes) in each group and all were women. The patients with SS and control subjects were comparable in the terms of age (the mean age of SS was 55.17 ± 9.68 years and the mean age of the control was 54.50 ± 9.25 years, *p* = 0. 808). The mean time since diagnosis in the SS group was 3.92 ± 1.93 years, mean ESSDAI was 5.75 (range: 1–18), IgG was 16.11 ± 6.74 g/L, complement C3 was 0.85 ± 0.14 g/L, complement C4 was 0.21 ± 0.08 g/L. Patients with SS had a significantly higher HADS score than controls (8.08 ± 3.65 vs. 2.58 ± 0.97; *p* < 0.001) (Table 1).

**Table 1:** General information of patients with SS and healthy subjects.

	**SS (*n =* 12,24 eyes)**	**HC (*n =* 12,24 eyes)**	***t***	***P*-value**
Age (y)	55.17 ± 9.68	54.50 ± 9.25	−0.244	0.808^a^
Gender
Female	12	12		1.0^b^
Duration of SS(y)	3.92 ± 1.93	N/A		
ESR (mm)	17.08 ± 8.43	N/A		
CRP (10mg/L)	2.73 ± 2.35	N/A		
ANA,n(%)	11(91.67)	N/A		
Anti SSA/Ro, n(%)	9(75.00)	N/A		
Anti SSB/La, n(%)	7(58.33)	N/A		
IgG (g/L)	16.11 ± 6.74	N/A		
C3 (g/L)	0.85 ± 0.14	N/A		
C4 (g/L)	0.21 ± 0.08	N/A		
ESSDAI—mean(range)	5.75(1-18)	N/A		
Salivary gland biopsy, *n* (%)	5(41.67)	N/A		
Focus Criteria + *n* (%)	2(40.00)	N/A		
Systolic blood pressure (mm Hg)	126.92 ± 5.60	123.08 ± 4.34	1.83	0.081^a^
Diastolic blood pressure (mm Hg)	78.67 ± 6.56	83.33 ± 6.82	1.671	0.109^a^
HADS	8.08 ± 3.65	2.58 ± 0.97	−7.139	**<0.001** ^a^

*Bold values indicates P <0.05*.

a *Independent t-test*.

b *Chi-square test*.

*SS, Sjogren's syndrome; HC, healthy control; ANA, antinuclear antibody; ESR, erythrocyte sedimentation rate; CRP, C-reactive protein; ESSDAI, EULAR SS disease activity index for disease activity; HADS, hospital anxiety and Depression Scale; N/A, not applicable*.

The SS group had lower VA than the control group (*p* < 0.001) and had shorter BUT (4.67 ± 0.92 vs. 13.42 ± 1.50 s; *p* < 0.001), OSS (3.12 ± 1.90 vs. 0; *p* < 0.0001), lower SIT score (3.33 ± 1.86 vs. 12.92 ± 1.32 mm; *p* < 0.001), and lower TMH (0.15 ± 0.04 vs. 0.58 ± 0.12 mm; *p* < 0.001) (Table 2).

**Table 2:** Ocular information of patients with SS and healthy subjects.

	**SS**	**HC**	***P*-value^a^**
	**(*n =* 12,24 eyes)**	**(*n =* 12,24 eyes)**	
VA (logMAR)	0.20 ± 0.14	0.05 ± 0.07	**<0.001**
Spherical equivalent	1.75 ± 0.25	1.50 ± 0.50	0.891
Mean IOP (mm Hg)	14.63 ± 1.58	15.13 ± 1.61	0.357
BUT(s)	4.67 ± 0.92	13.42 ± 1.50	**<0.001**
OSS	3.12 ± 1.90	0	**<0.001**
SIT(mm)	3.33 ± 1.86	12.92 ± 1.32	**<0.001**
TMH(mm)	0.15 ± 0.04	0.58 ± 0.12	**<0.001**

*Bold values indicates P <0.05*.

a *P-value was obtained with generalized estimating equation (both eyes of the subjects were included)*.

*SS, Sjogren's syndrome; HC, healthy control; VA, Visual acuity; IOP, intraocular pressure; BUT, Tear breakup time; OSS, Ocular staining score; SIT, Schirmer test; TMH, Tear meniscus height*.

### Macular RT

The subregional RT in the SS and control groups is shown in Table 3, Figure 1B. After adjusting for age, IOP, VA, and blood pressure (BP), inner RT was significantly lower in the SS group than in controls in the superior quadrant of the inner ring (*p* = 0.022) (Figure 1C). The other 3 regions of the inner ring (nasal, *p* = 0.739; inferior, *p* = 0.115; and temporal, *p* = 0.841), 4 regions of the outer ring (superior, *p* = 0.348; nasal, *p* = 0.161; inferior, *p* = 0.513; and temporal, *p* = 0.472), and foveal center (*p* = 0.244) did not differ significantly between groups.

**Table 3:** Comparison of macular retinal thickness at different locations between patients with SS and healthy subjects.

**Location**	**SS (*n =* 12,24 eyes)**	**HC (*n =* 12,24 eyes)**	***P*-value^a^**
**Macular inner retinal thickness (μm, mean** **±SD)**			
IS	107.88 ± 9.35	113.25 ± 5.82	**0.022**
OS	106.67 ± 12.26	111.21 ± 6.24	0.348
IN	105.88 ± 11.48	115.62 ± 6.60	0.739
ON	118.79 ± 9.34	123.50 ± 4.85	0.161
II	109.75 ± 11.16	114.25 ± 6.07	0.115
OI	102.29 ± 12.21	105.17 ± 6.31	0.513
IT	101.92 ± 18.93	104.92 ± 4.56	0.841
OT	91.62 ± 15.13	94.75 ± 5.29	0.472
C	47.46 ± 16.61	51.83 ± 5.65	0.244
**Macular outer retinal thickness (μm, mean** **±sd)**			
IS	210.63 ± 26.32	218.00 ± 5.33	0.118
OS	190.00 ± 26.29	191.54 ± 11.00	0.493
IN	202.58 ± 22.08	216.58 ± 11.82	0.581
ON	177.33 ± 19.50	195.71 ± 9.10	**<0.001**
II	209.92 ± 21.32	211.63 ± 7.53	0.228
OI	178.96 ± 31.61	181.25 ± 12.94	0.123
IT	214.87 ± 40.33	213.46 ± 9.01	0.302
OT	192.42 ± 36.15	193.92 ± 10.66	0.179
C	188.25 ± 23.10	193.29 ± 15.69	0.34
**Macular full retinal thickness (μm, mean** **±sd)**			
IS	318.50 ± 31.84	331.25 ± 8.06	0.794
OS	296.67 ± 35.68	302.75 ± 11.22	0.25
IN	308.46 ± 27.48	332.21 ± 15.07	0.487
ON	296.13 ± 23.17	319.21 ± 9.71	**0.007**
II	319.67 ± 28.48	325.88 ± 10.06	0.878
OI	281.25 ± 39.00	286.42 ± 13.16	0.253
IT	316.79 ± 53.82	318.38 ± 8.10	0.521
OT	284.04 ± 47.27	288.67 ± 11.75	0.423
C	235.71 ± 37.04	245.13 ± 17.04	0.259

*Bold values indicates P <0.05*.

a *Generalized estimating equation models were used to obtain P-values comparing mean inner,outer and full macular retinal thickness between SS patients and healthy subjects. Models were adjusted for age, intraocular pressure, acuity, blood pressure*.

*SS, Sjogren's syndrome; HC, healthy control; IS, inner superior; OS, outer superior; IN, inner nasal; ON, outer nasal; II, inner inferior; OI, outer inferior; IT, inner temporal; OT, outer temporal; C, center*.

Compared with healthy subjects, the outer RT of patients with SS was significantly lower in the outer nasal (ON) (*p* < 0.001) quadrants (Figure 1D). The other 3 regions of the outer ring (superior, *p* = 0.493; inferior, *p* = 0.123; and temporal, *p* = 0.179), the 4 regions of the inner ring (superior, *p* = 0.118; nasal, *p* = 0.581; inferior, *p* = 0.228; and temporal, *p* = 0.302), and foveal center (*p* = 0.340) showed no significant difference between groups.

The full RT in the nasal region of the outer ring was significantly thinner in patients with SS than in control subjects (*p* = 0.007) (Figure 1E). No significant differences between groups were observed in any of the other regions (*p* > 0.05).

In the univariate regression analysis, macular RT was negatively correlated with VA (*b* = −14.237, *p* < 0.001) but not with age, IOP, or BP. The multivariate regression analysis showed that advancing age (*b* = −0.149, *p* = 0.030) and poor VA (*b* = −17.758, *p* = 0.002) were significantly associated with thinner macular RT (Table 4).

**Table 4:** Univariate and multivariate regression analyses of association between macular retinal thickness with demographic and ocular parameters in patients with SS.

**Parameters**	**Univariate regression analysis Regression coefficient (β ±SE)**	***P*-value^a^**	**Multivariate regression analysis Regression coefficient (β ±SE)**	***P*-value^a^**
Age (y)	−0.008 ± 0.044	0.861	−0.149 ± 0.069	**0.030**
VA (logMAR)	−14.237 ± 3.930	**<0.001**	−17.758 ± 5.841	**0.002**
Mean IOP (mm Hg)	0.204 ± 0.217	0.348	−0.013 ± 0.287	0.965
Systolic blood pressure (mm Hg)	−0.178 ± 0.114	0.120	−0.200 ± 0.110	0.070
Diastolic blood pressure (mm Hg)	0.108 ± 0.072	0.136	0.206 ± 0.127	0.105

*Bold values indicates P <0.05*.

a *P-value was obtained with generalized estimating equation*.

*SS, Sjogren's syndrome; HC, healthy control; VA, Visual acuity; IOP, intraocular pressure*.

### Superficial Macular Retinal VD

**Figure 2:**
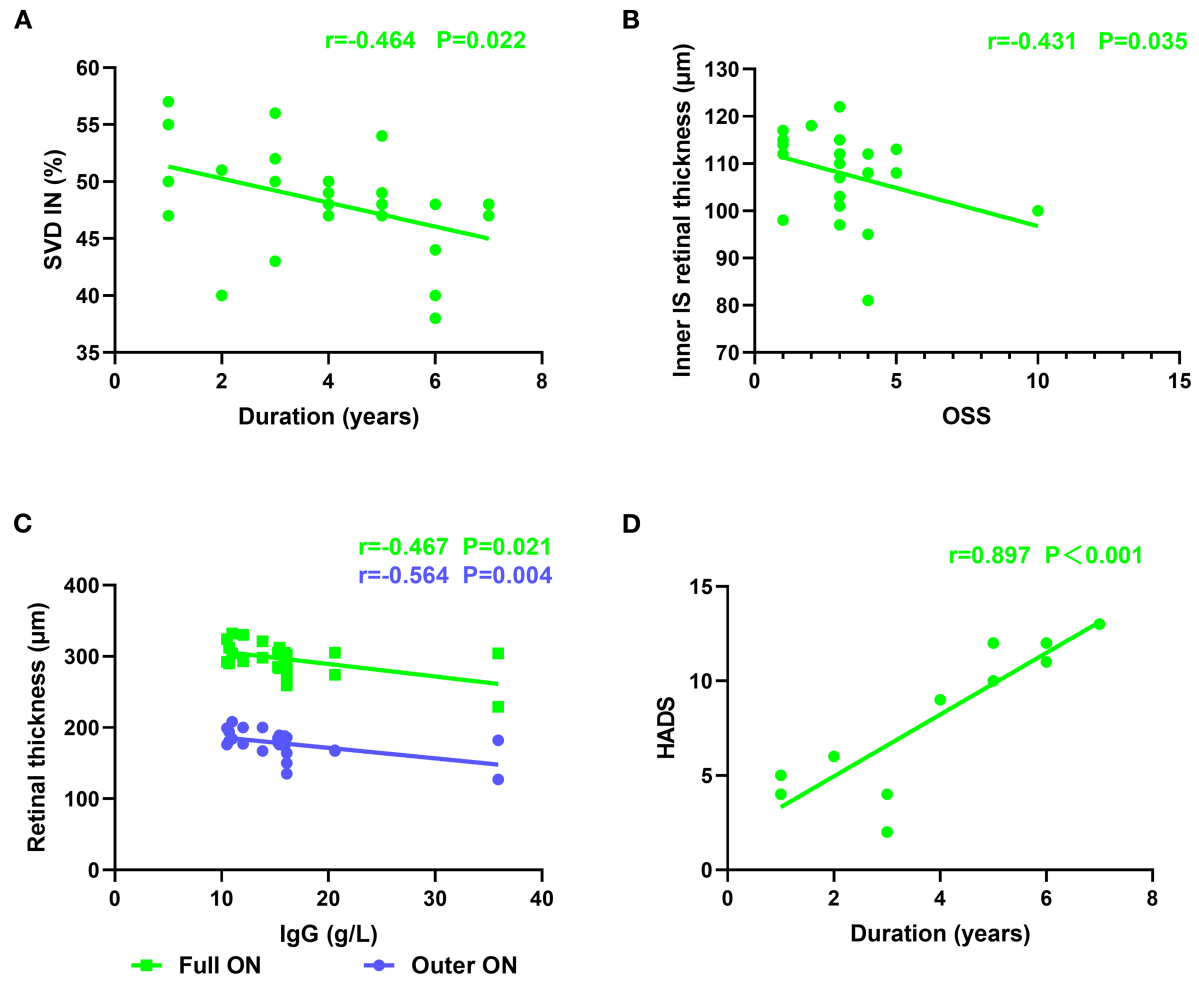
Retinal thickness was correlated with serum IgG level and OSS. The correlation between the duration of disease and HADS, SVD. **(A)** Negative correlation was found between duration and SVD at IN region (*r* = −0.464, *p* = 0.022). The vertical coordinate is the value of SVD, and the horizontal coordinate is the duration. **(B)** Negative correlation was found between RT and OSS in the inner retina at IS region (*r* = −0.431, *p* = 0.035). The vertical coordinate is the value of RT, and the horizontal coordinate is the value of OSS. **(C)** Negative correlation was found between RT and serum IgG level in the outer retina at ON region (*r* = −0.564, *p* = 0.004). Negative correlation was found between RT and serum IgG level in the full retina at ON region (*r* = −0.467, *p* = 0.021). The vertical coordinate is the value of RT, and the horizontal coordinate is the value of IgG. **(D)** Positive correlation was found between duration and HADS (*r* = 0.897, *p* < 0.001). The vertical coordinate is the value of HADS, and the horizontal coordinate is the duration. SS, Sjogren's syndrome; OSS, ocular staining score; HADS, hospital anxiety and Depression Scale; RT, retinal thickness; SVD, superficial vessel density; IS, inner superior.

Superficial VD at different retinal subregions in the SS and control groups is shown in Table 5, Figure 1B. After adjusting for age, IOP, VA, and BP, SVD was significantly lower in patients with SS than in controls on the nasal side (inner ring, *p* = 0.010 and outer ring, *p* = 0.007) and temporal region (inner ring, *p* = 0.048) (Figure 1F). In the SS group, SVD was negatively correlated with disease duration (−0.464) (Figure 2A).

**Table 5:** Comparison of superficial vessel density at different locations between patients with SS and healthy subjects.

**Location**	**SS**	**HC**	***P*-value^a^**
**(%, mean ±SD)**	**(*n =* 12,24eyes)**	**(*n =* 12,24eyes)**	
IS	49.58 ± 5.00	54.29 ± 4.44	0.093
OS	49.54 ± 4.20	52.58 ± 3.32	0.825
IN	48.25 ± 4.85	53.08 ± 4.52	**0.01**
ON	52.33 ± 4.97	56.17 ± 1.99	**0.007**
II	51.29 ± 5.90	53.62 ± 2.93	0.214
OI	50.17 ± 5.49	53.00 ± 3.40	0.59
IT	52.37 ± 7.22	54.75 ± 2.92	**0.048**
OT	48.38 ± 10.08	50.88 ± 2.89	0.291
C	16.58 ± 7.79	21.96 ± 3.16	0.916

*Bold values indicates P <0.05*.

a *Generalized estimating equation models were used to obtain P-values comparing mean superficial vessel density between SS patients and healthy subjects. Models were adjusted for age, intraocular pressure, acuity, blood pressure. SS, Sjogren's syndrome; HC, healthy control; IS, inner superior; OS, outer superior; IN, inner nasal; ON, outer nasal; II, inner inferior; OI, outer inferior; IT, inner temporal; OT, outer temporal; C, center*.

### ROC Curve Analysis of RT and SVD

**Figure 3:**
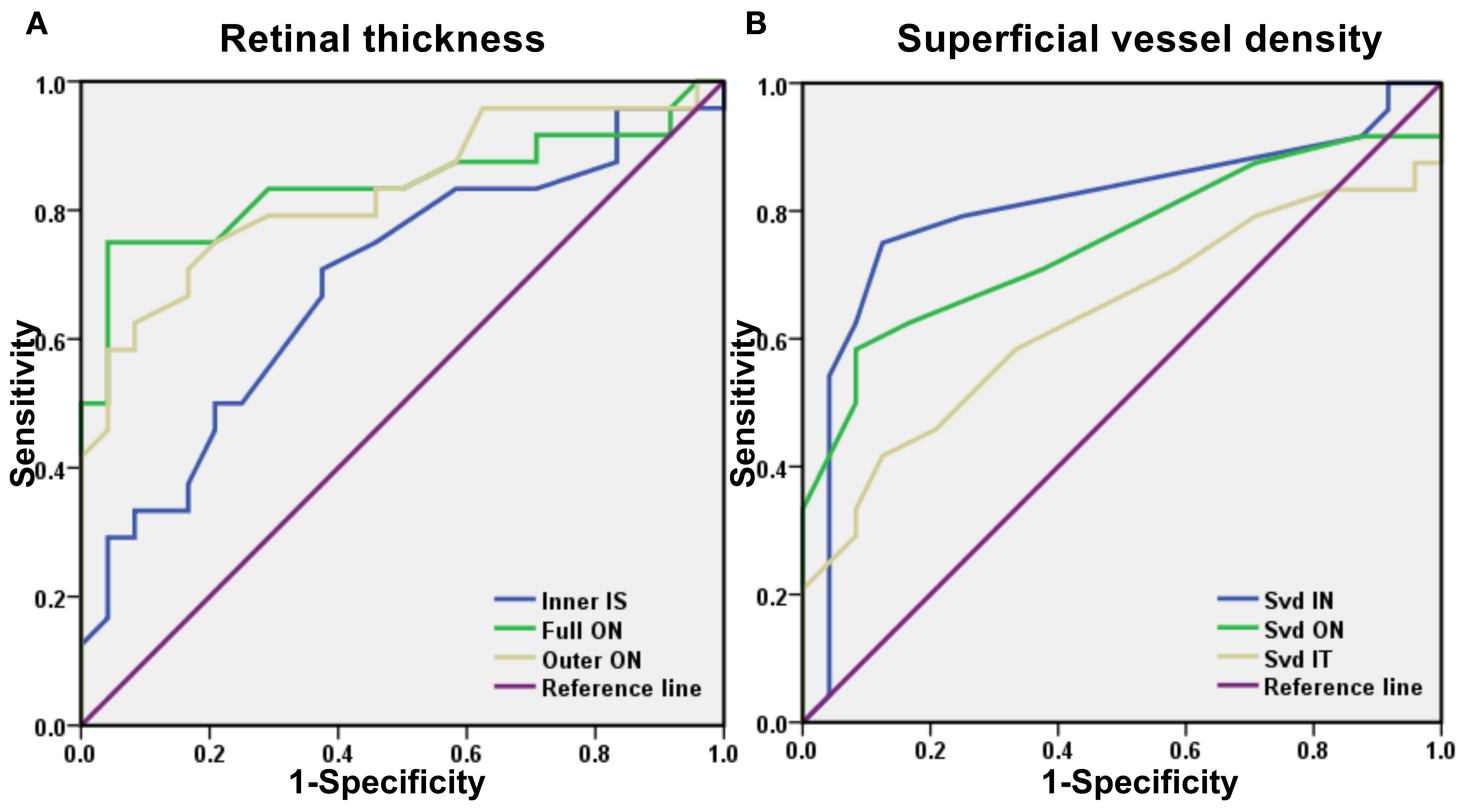
A receiver operating characteristic (ROC) curve analysis of RT and SVD. **(A)** The area under the ROC curve were 0.688 (95% *CI* = 0.536–0.839) for inner IS, full ON 0.839 (95% *CI* = 0.715–0.963), outer ON 0.828 (95% *CI* = 0.709–0.947). **(B)** The area under the ROC curve were 0.806 (95% *CI* = 0.671–0.942) for SVD IN, SVD ON 0.752 (95% *CI* = 0.607–0.896), SVD IT 0.635 (95% *CI* = 0.473–0.798). ROC, receiver operating characteristic; CI, confidence interval; RT, retinal thickness; SVD, superficial vessel density; ON, outer nasal; IT, inner temporal; IS, inner superior; IN, inner nasal.

Optical coherence tomography angiography data were analyzed to evaluate the specificity and sensitivity of RT and SVD as the diagnostic indicators of SS-related changes (Figure 3). Significant differences between groups were found in the inner superior (IS), outer ON, and full ON regions in the SS group. In the ON region, the area under the ROC curve for outer RT was 0.828 (95% *CI*: 0.709–0.947) and full RT was 0.839 (95% *CI*: 0.715–0.963), suggesting moderate to high diagnostic sensitivity for SS (Figure 3A).

Significant between-groups differences in SVD were found in the inner nasal (IN) and ON and inner temporal (IT) regions. The area under the SVD IN ROC curve was 0.806 (95% *CI*: 0.671–0.942), and the area under the SVD ON ROC curve was 0.752 (95% *CI*: 0.607–0.896), suggesting that SVD has moderate diagnostic sensitivity for SS (Figure 3B).

### Relationship Between RT, SVD, IgG, and OSS

**Figure 4:**
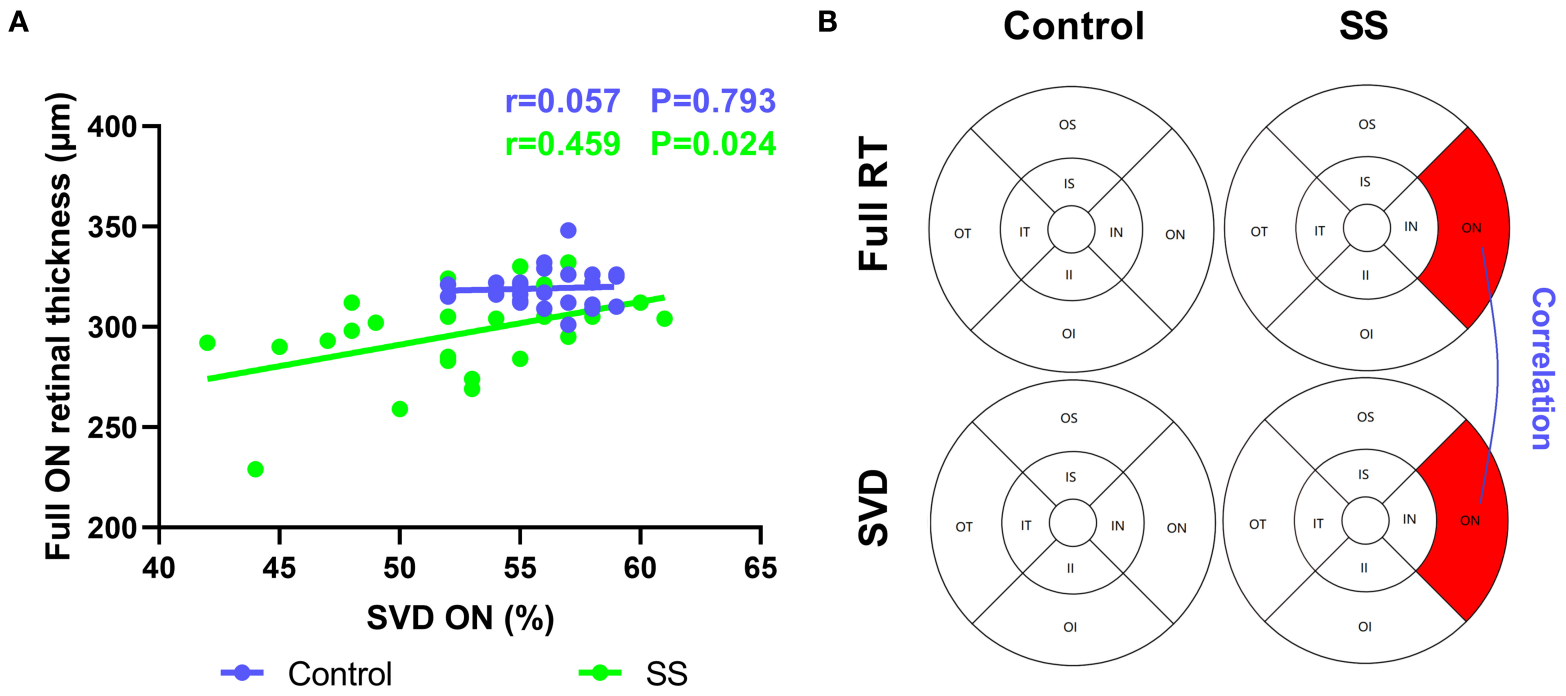
Correlation between RT and SVD in normal and patients with SS. The vertical coordinate is the value of RT, and the horizontal coordinate is the value of SVD. **(A,B)** In the SS group, SVD was positively correlated with full RT at ON region (*r* = 0.459, *p* = 0.024). SS, Sjogren's syndrome; RT, retinal thickness; SVD, superficial vessel density; ON, outer nasal.

In patients with SS, full RT was correlated with SVD in the ON region (*r* = 0.459) (Figure 4), suggesting that retinal thinning is related to decreased VD in SS. The inner RT in the IS region of the SS group was negatively correlated with OSS (−0.431), implying that a thinner RT is related to the greater severity of xerophthalmia (Figure 2B). In patients with SS, negative correlations were found between RT and serum IgG level in the outer and full retina at ON regions (−0.564 and −0.467, respectively) (Figure 2C).

### Relationship Between Disease Duration and HADS

A longer disease course in patients with SS was associated with a higher HADS index (0.897) (Figure 2D).

## Discussion

Sjogren's syndrome is characterized by exocrine gland involvement, abnormal proliferation of B lymphocytes, tissue infiltration of lymphocytes, and autoantibody production. The main clinical features dry eye, dry mouth, low fever, fatigue, and joint pain; about one-quarter of patients have visceral involvement. The risk of developing non-Hodgkin lymphoma is 40 times higher in patients with SS than in the general population (2). The EULAR and ACR issued a new classification standard for SS in 2016 (10) that emphasizes the pathologic features of the disease and the presence of autoantibodies; various indices corresponding to histology (labial gland biopsy), clinical indicators (SIT, OSS, and salivary flow rate), and immunology (anti–SS-A antibody) are stratified and weighted, and SS is diagnosed if the total score is ≥4. However, the lack of emphasis on the eyes in the assessment means that the significance of extraglandular changes in ophthalmic parameters are overlooked, although they are an important indicator of disease activity. We found that in the absence of clear ocular symptoms, patients in the SS group had significantly lower VA than healthy subjects, which has not been adequately addressed by other studies.

Sjogren's syndrome is referred as “benign lupus erythematosus” (34), which is another diffuse connective tissue disease associated with multiorgan damage; when the disease is active, the manifestations are similar to those of SLE and involve the vascular and nervous systems. The retina allows direct visualization of vascular changes (35) and provides a basis for early differentiation of patients with nervous system diseases (36). In our study, RT was calculated according to the ETDRS partition method and was found to be decreased in all areas of the retina in patients with SS, such as in the IS area of the inner retina, the ON area of the outer retina, and the ON area of the full-thickness retina. The results of the multiple regression analysis showed that visual impairment was associated with retinal thinning. This is in agreement with previous studies demonstrating that the ganglion cell inner plexiform and retinal nerve fiber layers were thinner in patients with SS compared with healthy subjects (37, 38). In healthy subjects, the inner RT is negatively correlated with age (39), which may be due to the loss of neurons, while the outer RT (especially RPE) may thicken with age (40). Our study found that full RT was negatively correlated with advancing age in patients with SS, this may need further longitudinal follow-up to confirm. The measurement of the full thickness of the retina in SS by OCTA has not yet been reported.

The choroidal blood supply is the most abundant in the body per unit weight and is therefore susceptible to inflammation in systemic diseases. Immune activation, autoantibody production, and impaired cellular immunity can damage the choroid. The activation of platelets and clotting pathways following vascular injury can lead to intravascular microthrombosis. The resultant vascular lesions and intimal hyperplasia of small arterioles followed by lumen stenosis lead to tissue hypoxia and chronic ischemia (41). The choroid is particularly sensitive to subclinical disease activity. In SLE, changes in the choroid may reflect reversible nephropathy and neuropathy (42). Similar changes in the retina may have clinical value for SS.

**Figure 5:**
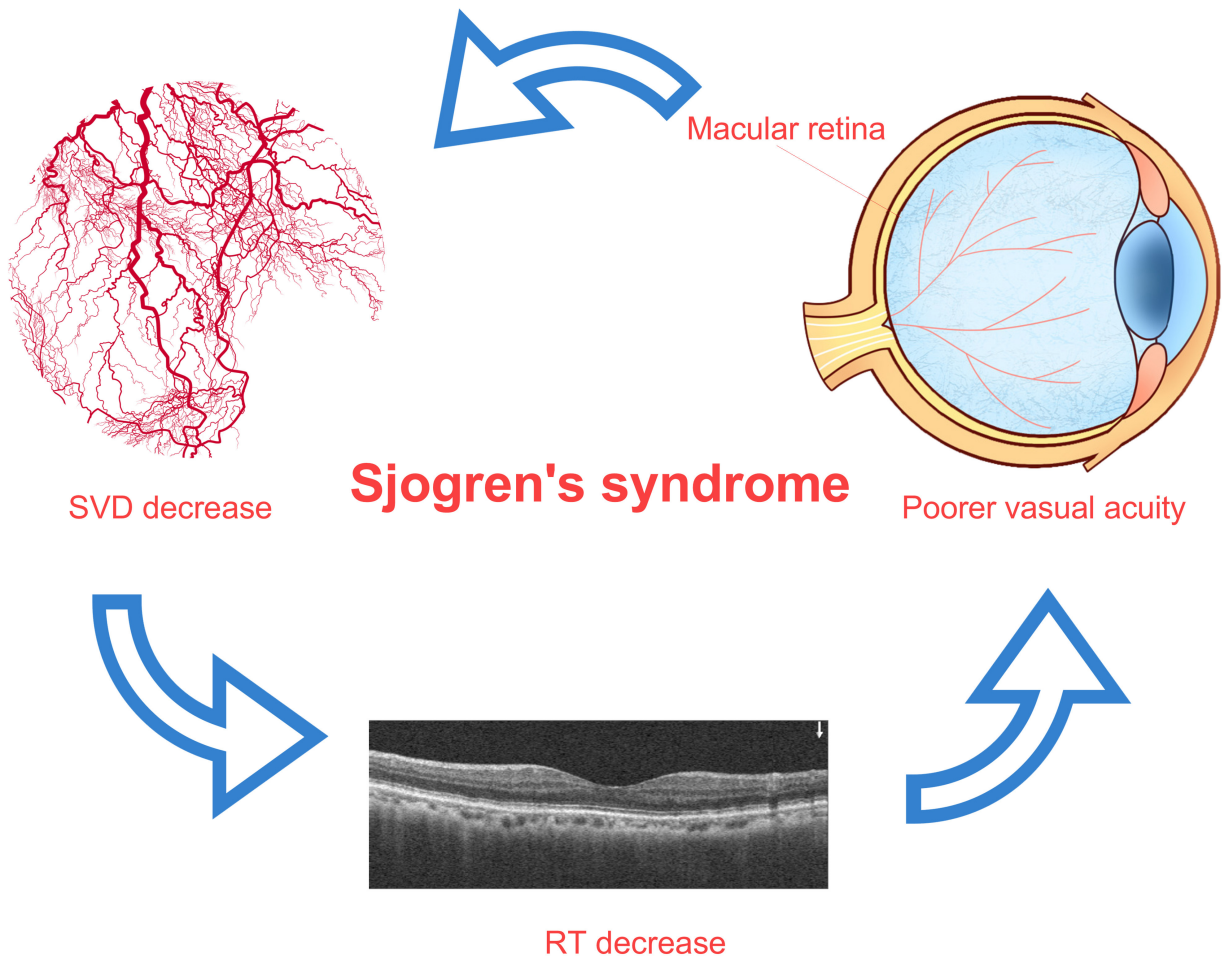
Relationship among SVD decrease, RT thinning, and visual impairment in patients with SS. In patients with SS, the decrease of superficial retinal vascular density in macular area may lead to the decrease of retinal thickness in related areas, and the decrease of retinal thickness in macular area may lead to the decrease of vision. The macular area is marked on the image of eyeball, which indicates the decrease of superficial vascular density in this area. SS, Sjogren's syndrome; RT, retinal thickness; SVD, superficial vessel density.

The retina and brain originate from the same embryonic tissue and both have comparable metabolic activity. A decrease in retinal blood supply can lead to the death of retinal cells (43). The internal blood flow of the retina is mainly supplied by the central retinal artery with the contribution from choroid vessels. The choroid can increase the partial pressure of O_2_ in the retina through the Root effect (44). When this occurs in the blood vessels supplying the retina, the resultant pathologic changes can affect the entire chorioretinal vascular network. Our study found that in patients with SS, the SVD showed a downward trend in all subregions with statistically significant decreases in the IN, ON, and IT regions. An increased neovascularization and the upregulation of vascular endothelial growth factor (VEGF)-A and its receptor VEGFR-2, which promote neovascularization, have been previously observed in the salivary glands of patients with SS (45), and angiogenesis is closely linked to the progression of SS (46). In contrast, we observed a decrease vascular density in the retina with increasing disease severity. Moreover, full RT was positively associated with SVD. Similar changes and associations have been reported in patients with diabetes (47) and Behcet's syndrome (18); alterations in microcirculation in these patients may precede clinically distinguishable retinopathy. We speculate that such subclinical changes exist in patients with SS. Retinal thinning was more obvious on the nasal side, while capillary loss was detected in the nasal and temporal regions. Choroidal capillary network lesions directly affect the blood supply to the outer retina; this can lead to chronic ischemia, especially of the photoreceptor layer and the death of rod cells and cone cells due to a decreased energy supply and loss of vision (43), which is supported by our findings (Figure 5).

The proliferation of peripheral B cells plays an important role in the development of SS. The overactivation of B cells is the main factor of hyperglobulinemia and autoantibody production (48, 49). At the same time, the disorder of peripheral B cell subsets exists in SS, such as the significant decrease of CD27 + memory B cells, the increase of CD27 initial B cells and CD19 + B cells (50), and the positive correlation between the number of CD19 + B cells and IgG serum level and hypergammaglobulinemia (51). In SLE, the deposition of IgG immune complex in retinal vascular wall is associated with retinal nerve fiber layer infarction and ganglion cell atrophy (52). In patients with glaucoma, the loss of retinal ganglion cells is accompanied by the accumulation of IgG autoantibodies on ganglion cell layer cells (53), and the deposition of IgG autoantibodies is accompanied by CD27 +/IgG + plasma cells, which occurs under pro-inflammatory conditions, the level of tumor necrosis factor-α (TNF-α), interleukin (IL)-6, and IL-8 were also increased (53). Immunity leads to antigen-specific and complex systemic immune responses, including the production of auto reactive antibodies against retinal and optic nerve epitopes with an increasing and time-dependent severity (53). Autoantibodies against a variety of retinal antigens have been reported, such as recoverin, α-Enolase, heat shock protein, arrestin, transducing protein, neurofilament protein, carbonic anhydrase II, and Tubby-related protein 1 (TULP1) (54). In our study, RT was found to be negatively correlated with IgG, indicating that RT may be affected by the abnormal immune state of SS disease. The exact mechanism of vascular occlusion in autoimmune diseases is still unclear, however, some possible pathogenic mechanisms include immune complex deposition, complement activation with microvascular thrombosis, and the fibrinoid degeneration of vascular wall (55). This may also be the possible reason for the decrease of SVD in patients with SS, and indirectly lead to the thinning of RT.

In patients with SS, an imbalance in the ratio of type 1 to type 2 helper T cells (56) leads to excessive interferon-γ (57), IL-17 (58), IL-21 (59), and IL-22 (60) production, which creates an inflammatory microenvironment that can cause tissue damage. Goblet cells are the main cells responsible for eye lubrication and their secretory ability is an important indicator of eye surface health. The secretory function and proliferative capacity of goblet cells are inhibited by increased inflammatory cytokine levels, resulting in changes in the ocular surface and irregular tear secretion (61). The OSS, similar to SIT test and BUT, is a reliable and objective index of the degree of dryness of the eyes. Our results showed that with the worsening of dry eye, the SIT score and BUT decreased while OSS increased. Additionally, SIT, BUT, and TMH were reduced whereas OSS was significantly higher in patients with SS compared with control subjects while inner RT was negatively correlated with OSS, which indirectly reflected the degree of dryness of the eyes.

Mental health disorders are more common in patients with SS than in healthy individuals (62, 63). The prevalence of anxiety and depression in patients with SS in China is 33.8 and 36.9%, respectively (64). Anxiety and/or depression are associated with lower physical activity and treatment compliance, which can have adverse effects on the well-being of patients with SS and may increase the incidence rate of cardiovascular disease and exacerbate SS (65). In our study, patients with SS had a higher HADS score than control subjects, which was positively correlated with disease duration.

The results of the ROC curve analysis of outer and full RT in the ON region indicated that these parameters have diagnostic utility for SS. Early diagnosis and accurate evaluation are critical for the successful treatment and good prognosis. SS has variable clinical manifestations, disease course, and prognosis, and clinical symptomatology is often unrelated to the degree of gland destruction. Classification criteria are standardized tools for the selection of appropriately defined homogeneous patient groups for research; however, they often fail to identify atypical individuals (20). OCTA is a noninvasive and convenient imaging method that can provide information on intraocular vascular network perfusion; RT measured by OCTA is a potential biomarker that can aid the diagnosis of SS. However, there is no previous report on the use of OCTA in patients with SS. So we did a preliminary study prudently. We set up a control group of healthy subjects in this study, so that we can distinguish SS from healthy people first through OCTA and then we will set up other control groups to test whether OCTA is helpful to distinguish SS from other diseases. At present, we are only exploring a possibility. We will conduct additional studies with larger samples to validate its diagnostic utility. At this stage, OCTA may be used in addition to the existing classification criteria.

This study had some limitations. First, given that patients with HCQ-induced retinochoroidosis were excluded, we did not consider the role of HCQ in SS in detail. Second, as the sample size was small, our findings require validation in a larger cohort before they can be translated into clinical practice. Due to the obvious gender bias of SS, the current study recruits female patients. With the deepening of the study, we will include more cases, both male and female will be taken into account. Third, RT is affected by many factors (such as, age and spherical equivalent). We will consider the influence of related factors as much as possible in the future study.

## Conclusions

We used OCTA to evaluate the significance of RT and SVD in SS, and found that the inner, outer, and full RT were thinner while SVD was decreased in the ON, IN, and IT regions of patients with SS compared with controls; additionally, RT is positively correlated with SVD and negatively related to IgG. Thus, retinal thinning in the macular area—which affects vision—can also reflect the severity of dry eyes in SS and has clinical value for assisted imaging diagnosis.

## Data Availability Statement

The datasets presented in this study can be found in online repositories. The name of the repository and accession number can be found below: Figshare, https://figshare.com/s/793657ff2e10765b6616.

## Ethics Statement

The studies involving human participants were reviewed and approved by the Medical Ethics of the First Affiliated Hospital of Nanchang University (cdyfy2018026). Written informed consent for participation was not required for this study in accordance with the national legislation and the institutional requirements.

## Author Contributions

RL: writing the original draft and data analysis. YS: data analysis and editing. YW, QL, QX, TX, TH, SC, and SL: data collection. YS and RW: supervision and editing. All authors contributed to the article and approved the submitted version.

## Funding

This research is supported by The Central Government Guides Local Science and Technology Development Foundation (20211ZDG02003); Key Research Foundation of Jiangxi Province (No: 20181BBG70004); Excellent Talents Development Project of Jiangxi Province (20192BCBL23020); Natural Science Foundation of Jiangxi Province (20181BAB205034); Grassroots Health Appropriate Technology Spark Promotion Plan Project of Jiangxi Province (No: 20188003); the Health Development Planning Commission Science Foundation of Jiangxi Province (No: 20201032); and the Health Development Planning Commission Science TCM Foundation of Jiangxi Province (No: 2018A060).

## Conflict of Interest

The authors declare that the research was conducted in the absence of any commercial or financial relationships that could be construed as a potential conflict of interest.

## Publisher's Note

All claims expressed in this article are solely those of the authors and do not necessarily represent those of their affiliated organizations, or those of the publisher, the editors and the reviewers. Any product that may be evaluated in this article, or claim that may be made by its manufacturer, is not guaranteed or endorsed by the publisher.
